# Comprehensive Understanding of the Kinetic Behaviors of Main Protease from SARS-CoV-2 and SARS-CoV: New Data and Comparison to Published Parameters

**DOI:** 10.3390/molecules28124605

**Published:** 2023-06-07

**Authors:** Fangya Li, Tingting Fang, Feng Guo, Zipeng Zhao, Jianyu Zhang

**Affiliations:** School of Pharmaceutical Science and Technology, Tianjin University, Tianjin 300072, China; lifangya@tju.edu.cn (F.L.); fangtingting815@163.com (T.F.); guofengcr7@163.com (F.G.); zzp_2020@tju.edu.cn (Z.Z.)

**Keywords:** kinetic parameters, molecular docking, main protease, SARS-CoV-2

## Abstract

The main protease (M^pro^) is a promising drug target for inhibiting the coronavirus due to its conserved properties and lack of homologous genes in humans. However, previous studies on M^pro^’s kinetic parameters have been confusing, hindering the selection of accurate inhibitors. Therefore, obtaining a clear view of M^pro^’s kinetic parameters is necessary. In our study, we investigated the kinetic behaviors of M^pro^ from SARS-CoV-2 and SARS-CoV using both FRET-based cleavage assay and the LC-MS method, respectively. Our findings indicate that the FRET-based cleavage assay could be used for preliminary screening of M^pro^ inhibitors, while the LC-MS method should be applied to select the effective inhibitors with higher reliability. Furthermore, we constructed the active site mutants (H41A and C145A) and measured the kinetic parameters to gain a deeper understanding of the atomic-level enzyme efficiency reduction compared to the wild type. Overall, our study provides valuable insights for inhibitor screening and design by offering a comprehensive understanding of M^pro^’s kinetic behaviors.

## 1. Introduction

The human coronavirus pandemic has been disturbing the life and economy of the world in recent years, especially the COVID-19 disease, with more than six million people dying as a result of SARS-CoV-2. Efforts to curb the spread of this virus have included extensive research on coronavirus infection. These studies have focused on understanding important protein functions [1,2,3,4], structure analysis [5,6,7], inhibitor selection, and drug design [8,9,10,11]. The coronavirus has a large genome (25–32 kb) containing two overlapping open reading frames (ORF1a and ORF1b) that encode two polyproteins, pp1a (490 kDa) and pp1ab (794 kDa) [12,13,14]. The polyproteins are processed by papain-like protease (PL^pro^) and main protease (M^pro^, also named 3C-like protease, 3CL^pro^). It is reported that the main protease (M^pro^) plays a crucial role in replication, transcription, and modification in virus life by cleaving the polyproteins into sixteen non-structural proteins [15,16]. Furthermore, M^pro^ is highly conserved among human coronaviruses, with a low possibility of mutation, and has no homologous gene in humans [17,18,19]. Hence, M^pro^ has become an attractive and perfect target for antiviral drug development. Tremendous studies of M^pro^ in the aspects of protein functions [1,20,21], crystal structures [18,22,23], kinetic parameters study [24,25,26,27], inhibitor screening and selection [28,29], and related drug design have been reported [30,31]. One of the most important processes is the achievement of various inhibitors for M^pro^, such as 11a [27], N3 [32], Carmofur [33] inhibitors (binding on the active site), and Pelitinib [34] (acting on allosteric 1 site), as well as the recent oral drugs nirmatrelvir [11] (PF-07321332) of M^pro^ from Pfizer. However, the basic parameters to characterize the activity of M^pro^ vary from group to group, leading to significant variations in the estimated values. For instance, the difference in the efficiency of protease (*k*_cat_/K_m_) can reach more than a thousandfold, with the *k*_cat_/K_m_ value of SARS-CoV-2-M^pro^ ranging from 28,500 M^−1^s^−1^ to 219 M^−1^s^−1^, even when measured using the same technique (FRET-based cleavage assay) [17,32]. Additionally, the different methods used to determine the activity of M^pro^ provide varying results, as seen in the *k*_cat_/K_m_ value of SARS-CoV-M^pro^, which is 6800 M^−1^s^−1^ using the FRET-based cleavage assay compared to 35.5 M^−1^s^−1^ using the LC-MS method [26,35]. These different results have turned the kinetic parameters of M^pro^ confusion. Previous studies synthesized different substrates and determined their hydrolysis activity by measuring the kinetic parameters and further explaining the specificity position of substrates [26]. Comparing the difference in catalytic efficiency between M^pro^ from SARS-CoV and MERS-CoV has provided a more comprehensive understanding of the target protein’s catalytic mechanism [36]. Moreover, by solving the complex structures of SARS-CoV-2 H41A and six peptides and detecting the binding affinity between them, the subsequence recognition and selective subsites of M^pro^ were determined [23]. Therefore, as a fundamental property of the enzyme, it is necessary to make a clear view of the kinetic behaviors of M^pro^. To address these discrepancies, we investigated the kinetic parameters of M^pro^ from SARS-CoV-2 and SARS-CoV using both the FRET-based cleavage assay and the LC-MS method. Our results suggest that using the FRET-based cleavage assay together with less accurate V_max_ values influences the catalytic efficiency of M^pro^. Furthermore, we also measured the kinetic behaviors of mutants H41A and C145A, which have lower enzyme activity, to provide a molecular-level view to understand the kinetic behavior. Our study on the kinetic behaviors of M^pro^ provides an overall view of the characterization of its activity and offers valuable insights for inhibitor screening and design.

## 2. Results and Discussion

### 2.1. Purification and Oligomerization State of M^pro^

**Figure 1:**
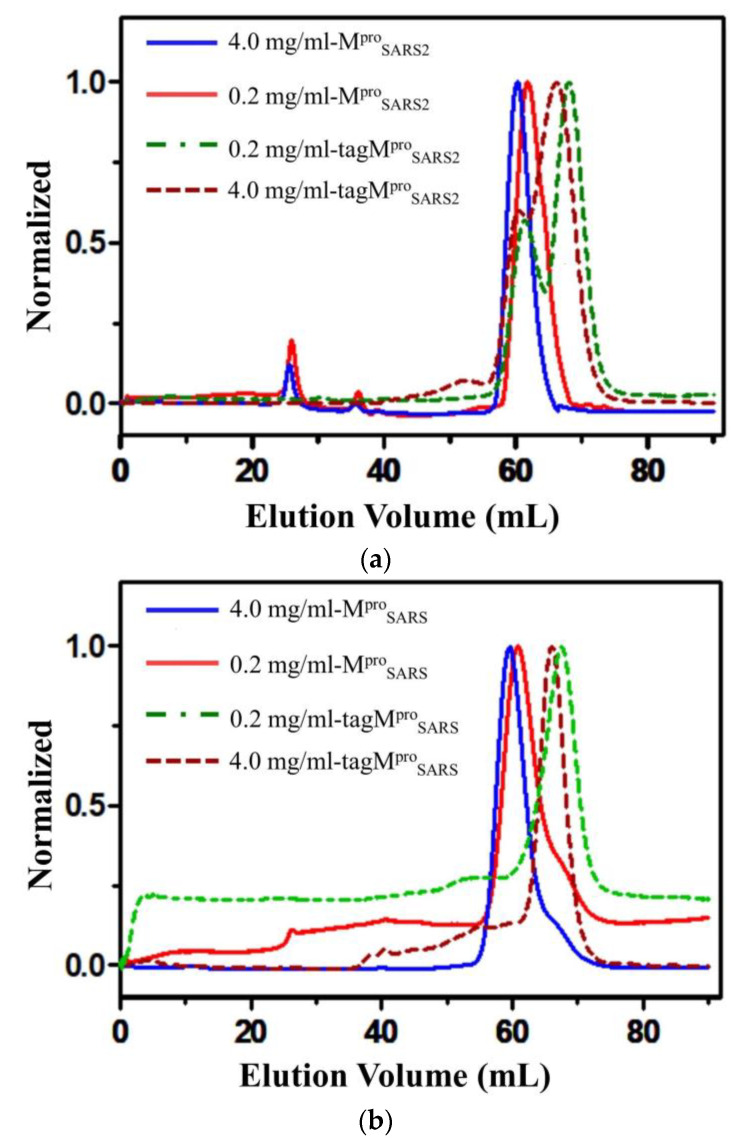
Gel filtration study of (**a**) M^pro^_SARS2_ and tagM^pro^_SARS2_ (tag_MAS_M^pro^_SARS2LE6×His_) and (**b**) M^pro^_SARS_ and tagM^pro^_SARS_ (tag_34aa_M^pro^_SARS_). The solid line (M^pro^) shows one peak corresponding to the dimer on the elution profile of gel filtration column chromatography. The dot-and-dash line (tagM^pro^) shows a major peak corresponding to the monomer. The Y-axis was normalized to the same height for better comparability.

The quaternary structure of a protein plays a critical role in determining its function [37,38]. Previous studies have demonstrated that the presence of additional amino acids at the N- or C-terminal might impact the state of M^pro^ (dimer or monomer), although the underlying mechanism remains unclear. Specifically, the extra amino acids at the N terminus have been shown to affect the dimerization of the M^pro^ and promote the formation of the monomeric state in solution [21,39,40]. To investigate the influence of oligomerization state on protein activity, we constructed both an authentic protein (marked as M^pro^) and a tagged protein (tagM^pro^). The target proteins were expressed in *E. coli* and purified, as illustrated in Figure S1. To identify the state of the target proteins, we performed analytical gel filtration of both M^pro^ and tagM^pro^ with low and high concentrations (0.2 mg/mL and 4 mg/mL) using Uniondex 75 pg 16/60 size column (Figure 1). Based on Figure 1, it is evident that there was only one early peak present, whether in low or high concentrations, suggesting that only dimer configurations were formed by the authentic main protease (M^pro^) from SARS-CoV-2 and SARS-CoV. For tag_MAS_M^pro^_SARS2LE6×His_ with both N- terminus and C- terminus extensions, two peaks were observed at both low and high protein concentrations, with higher late peaks, suggesting a mixture of dimer and monomer configurations, where the major form was the monomeric protein. There was only one peak to the corresponding monomer of tag_34aa_M^pro^_SARS_. The different oligomerization states between tag_MAS_M^pro^_SARS2LE6×His_ and tag_34aa_M^pro^_SARS_ may be attributed to the different number of amino acids at the N-terminal of M^pro^. tag_MAS_M^pro^_SARS2LE6×His_ had an additional three amino acids at the N-terminus, while tag_34aa_M^pro^_SARS_ had thirty-four_._ A previous study [35] suggested that increasing the number of additional residues at the N terminus would lead to a decreased enzyme activity, reflecting a lower possibility of forming the dimers of M^pro^. Therefore, it can be concluded that additional amino acids at the N- or C- terminus may interrupt the formation of dimers, which aligns with the prior general conclusions [35,41,42]. The previous study reported that the dimer or monomer state of M^pro^ depends on the concentration of protein: at the concentration of 4 mg/mL, SARS-CoV-M^pro^ was composed of both dimers and monomers; at low protein concentration (0.2 mg/mL), protein would form only monomers [42]. Our study here shows that the state of the target protein is concentration-independent.

### 2.2. The Kinetic Behaviors of M^pro^

Catalytical efficiency is a critical parameter for an enzyme, including the main protease targeted for treating human coronavirus. Despite extensive research on its function, there are limited reports on its detailed kinetic behavior. Furthermore, even using the same kinetic measurement method, different studies have reported significant variations in the observed kinetic behaviors of M^pro^ (Table 1 and Table 2).

**Figure 2:**
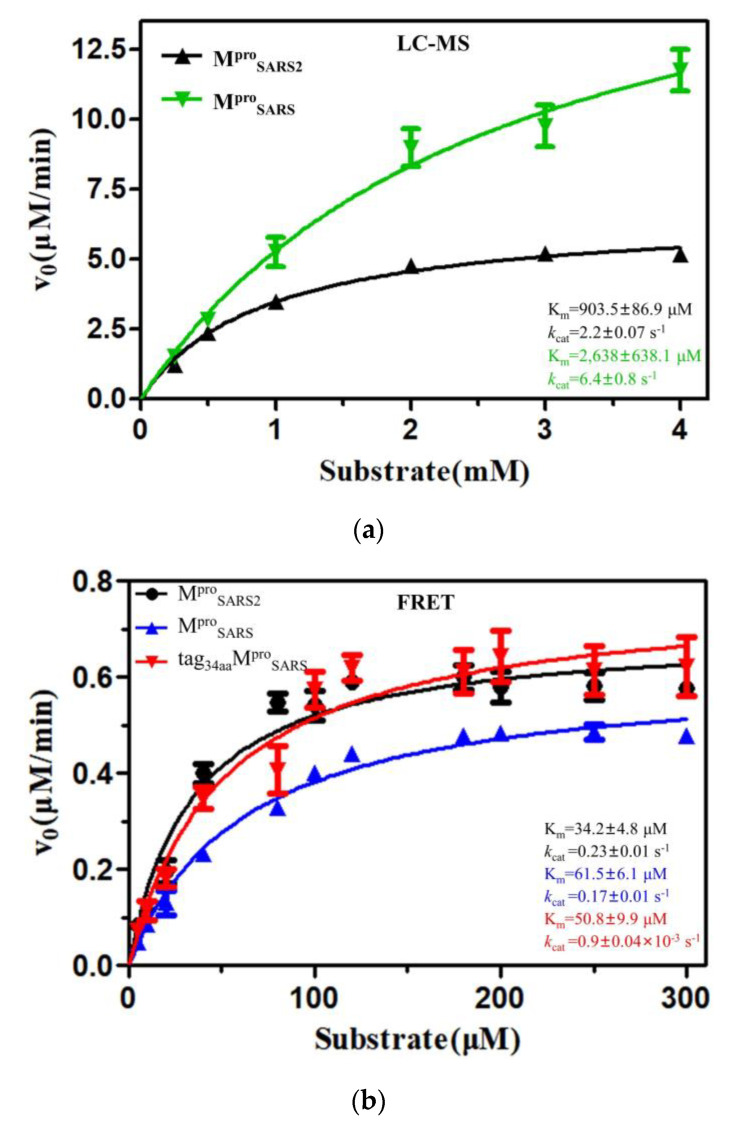

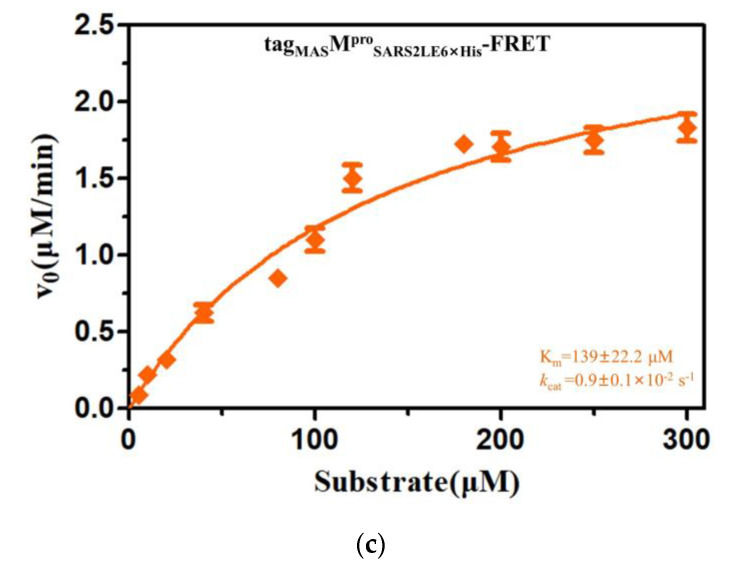
The Michaelis–Menten curve for M^pro^ from SAR-CoV-2 and SARS-CoV using different methods. (**a**) LC-MS method and (**b**,**c**) FRET-based cleavage assay. Values are means ± SEM of at least three independent experiments performed in triplicates. Curves fit with the Michaelis–Menten equation in GraphPad Prism.

The lack of consensus on M^pro^’s kinetic behavior limits the understanding of this enzyme. Moreover, the use of different forms of M^pro^ with varying activities in different studies raises important questions regarding the reliability of conclusions drawn from experiments, such as inhibitor screening and X-ray crystal studies, if the protein used is of the wrong form and has low activity. Therefore, to obtain a clear view of the M^pro^ activity, the kinetic behaviors of the target protein were investigated using the two most common methods (the FRET-based cleavage assay and LC-MS method) in our study (Figure 2, Figure S2 and Figure S3). To gain a thorough understanding of the catalytic efficiency of M^pro^, we summarized the kinetic results of M^pro^ with various forms and kinetic parameters reported previously using different methods in Table 1 (for SARS-CoV-2) and Table 2 (for SARS-CoV), allowing for easy comparison. With the acquisition of additional data using the FRET-based cleavage assay, we have prioritized the examination of the kinetic parameters from FRET. Notably, for M^pro^ from SARS-CoV-2, it is evident that the activity of the authentic form (M^pro^, *k*_cat_/K_m_ = 6800 ± 976 M^−1^s^−1^) is much higher than the tagged form (tag_MAS_M^pro^_SARS2LE6×His_, *k*_cat_/K_m_ = 67.5 ± 11.8 M^−1^s^−1^, Entry 10 and 11 in Table 1). A similar trend can be observed with the protease from SARS-CoV, exhibiting a difference of around 164-fold (Entry 15 and 16, Table 2) between M^pro^_SARS_ and tag_34aa_M^pro^_SARS_ (with longer extra amino acids at the N-terminal of tag protein). This suggested that extra amino acids at the N-terminal influenced the formation of dimerization and further reduced the enzyme activity of tagM^pro^. The previous study also reported that the kinetic parameters of M^pro^_SARS_, tagM^pro^-GP-6×his, and tagGPLGS-M^pro^ were 26,500, 6800, and 167 M^−1^s^−1^, respectively. The intrinsic reason may be attributed to the influence of Ser1 from the second protomer. The presence of additional amino acids at the N-terminal would disrupt the proper formation of Ser1, further affecting the conformation of the active site and contributing to decreased enzyme activity [35]. These results support that the N-terminal plays an important role in maintaining enzyme activity and that the dimer maintains the enzyme activity.

The LC-MS method is known for its complex procedures and high cost, resulting in a limited amount of data compared to the FRET-based cleavage assay. Specifically, the K_m_ value of M^pro^ measured by the LC-MS method is in the millimolar range, which is much higher than that obtained by the FRET-based cleavage assay, typically in the micromolar range (usually less than 200 μM). The *k*_cat_ and K_m_ values measured by the LC-MS method were higher than those measured by the FRET-based cleavage assay, while the *k*_cat_/K_m_ value was higher using the FRET-based cleavage assay (around 1- to 3-fold). For SARS-CoV-2, the *k*_cat_ and K_m_ values of M^pro^ measured by the LC-MS method were 9 and 24 times higher than that of using the FRET-based cleavage assay (Entries 10 and 16 in Table 1). This trend was observed not only for SARS-CoV-2 but also for the target protein from SARS-CoV (Entry 15 and 28 in Table 2). These results strongly suggest that the kinetic parameters of the same protease were different when measured using different methods. The K_m_ values measured by FRET are all lower than the values obtained by the LC-MS method. We consider that the FRET-based cleavage assay has its limitations; because of the intermolecular interactions between the Dabcyl group and the Edans group (free or in the substrate), fluorescence quenching may occur, further reducing the actual fluorescence intensity. This may influence the calculation of the V_max_ value and affect the accuracy of the kinetic parameters of M^pro^. This situation is not present in the LC-MS method. In addition to the quenching problem, the form of the substrate also influences the accuracy of the kinetic parameters of the target protein. Although the key amino acids in substrates were the same, the fluorophore was different. As Table 1 and Table 2 show, the *k*_cat_/K_m_ value of M^pro^ varied: the *k*_cat_/K_m_ value measured in Yang’s group [32] is 28,500 M^−1^s^−1^ while it is 5748 ± 1135 M^−1^s^−1^ in Wang’s study [43]. Because of the substrate with different fluorophores used in their studies, different *k*_cat_/K_m_ values of the same protease could occur. If the same substrate is used in different groups, a similar catalytic efficiency of M^pro^ could be observed: in our study, the *k*_cat_/K_m_ value of M^pro^_SARS2_ and M^pro^_SARS_ were 6800 ± 976 M^−1^s^−1^ and 2792 ± 293 M^−1^s^−1^, respectively, compared to 3426 ± 417M^−1^s^−1^ and 3011 ± 294 M^−1^s^−1^ in Rolf Hilgenfeld’s study [45]. Therefore, different fluorophores could cause different degrees of fluorescent molecular interactions and further influence the study of the kinetic behaviors of target proteins. Based on the LC-MS principle, enzyme activity measurements were conducted without any other external influences. On the other hand, it should be noted that only one assay is not adequate to fully reflect enzyme characteristics. Therefore, the kinetic parameters measured by the LC-MS method are more reliable. That is why the effect of some inhibitors was disappointing while the K_m_ (or K_d_) values were in the nanomolar range.

One of the important fields focusing on M^pro^ is the screening of inhibitors. To rapidly search for novel inhibitors of SARS-CoV-2 M^pro^, Emily et al. screened a library with high-content protease inhibitors against the main protease and obtained 27 hits, each with more than 50% inhibition, using a FRET assay [52]. Based on the fluorescence polarization (FP) technique and biotin–avidin system (BAS), a step-by-step sandwich-like FP screening assay was developed. It is a relatively quick identification of SARS-CoV-2 M^pro^ inhibitors from natural product libraries. Researchers identified Dieckol as a novel potential inhibitor against SARS-CoV-2 M^pro^ using the screening assay [53]. FRET substrates with a preference for 2-Abz/Tyr(3-NO_2_) FRET pairs were characterized, and identified two FRET substrates as promising candidates for screening and inhibitor characterization [54]. Therefore, we strongly recommend using fluorescence assays as a more convenient way to screen inhibitors, followed by further verification through the LC-MS method to target potent inhibitors.

Various residues can influence the enzyme activity of M^pro^, including the catalytic dyad (His41 and Cys145); amino acids involved in substrate binding (like Glu166 and His163); and residues related to dimerization (like Arg298 and Asn214). Mutating Glu166 to alanine resulted in decreased enzyme activity, with *k*_cat_/K_m_ of 877 ± 132 s^−1^M^−1^ compared to the *k*_cat_/K_m_ of the wild type, which was 2830 ± 303 s^−1^M^−1^. Once Arg298 was substituted by alanine, the *k*_cat_ was 0.10 ± 0.004 s^−1^ [55]. However, most studies did not describe the exact catalytic efficiency of mutants in active sites and only stated the relatively proteolytic activity of mutation at the catalytic dyad (His41 and Cys145) [50]. Therefore, to investigate the pre-cleavage state of SARS-CoV-2 M^pro^ [23,56], the mutant H41A and C145A were introduced. In our study, to further gather more information about the kinetic behaviors of M^pro^, the kinetic parameters of mutants (H41A and C145A) were also measured. As shown in Table 1, using the FRET-based cleavage assay, the catalytic efficiency of H41A (*k*_cat_/K_m_ = 292.1 ± 55.5 M^−1^s^−1^) and C145A (*k*_cat_/K_m_ = 319.3 ± 54.7 M^−1^s^−1^) from SARS-CoV-2 was approximately 21-fold lower than WT (*k*_cat_/K_m_ = 6800 ± 976 M^−1^s^−1^). Similarly, for the protease from SARS, the activity reduction ranged from 54 (C145A, *k*_cat_/K_m_ = 51.3 ± 6.9 M^−1^s^−1^) to 150 (H41A, *k*_cat_/K_m_ = 18.5 ± 3.7 M^−1^s^−1^) times compared to M^pro^_SARS_ (*k*_cat_/K_m_ = 2792 ± 293 M^−1^s^−1^) (Entry 15, 17 and 18 in Table 2). Furthermore, the catalytic efficiency of these mutants measured by the LC-MS method (7.4 ± 1.9 × 10^−2^ M^−1^s^−1^ for tag_GST_C145A_SARS2GP6×His_; 20.1 ± 4.1 × 10^−2^ M^−1^s^−1^ for tag_GST_H41A_SARS2GP6×His_) was lower than the one obtained by the FRET-based cleavage assay. These results indicated the importance of His41 and Cys145 in maintaining protease activity.

### 2.3. Molecular Docking of M^pro^ with Substrate

The kinetic behaviors of M^pro^ have been measured using both the FRET-based cleavage assay and the LC-MS method, respectively. Comparison of M^pro^’s different kinetical behaviors between different proteins is also possible. However, the detailed reasons that affect enzyme efficiency remain unclear. Thus, it is necessary to explore the inter-relationship between substrates and target proteins at the atomic level. Previous studies have identified six probable inhibitors against M^pro^_SARS2_ through molecular docking, and ADMET profile prediction has shown that the best-docked phytochemicals were safe and possessed drug-like properties [57]. Shilpa Das’s group has also determined that nigellidine exhibits hepato-reno-protective, antioxidant-immunomodulatory, and anti-inflammatory activities with inhibitory potential against COVID-19 proteins combined with molecular docking methods and experiments [58].

**Figure 3:**
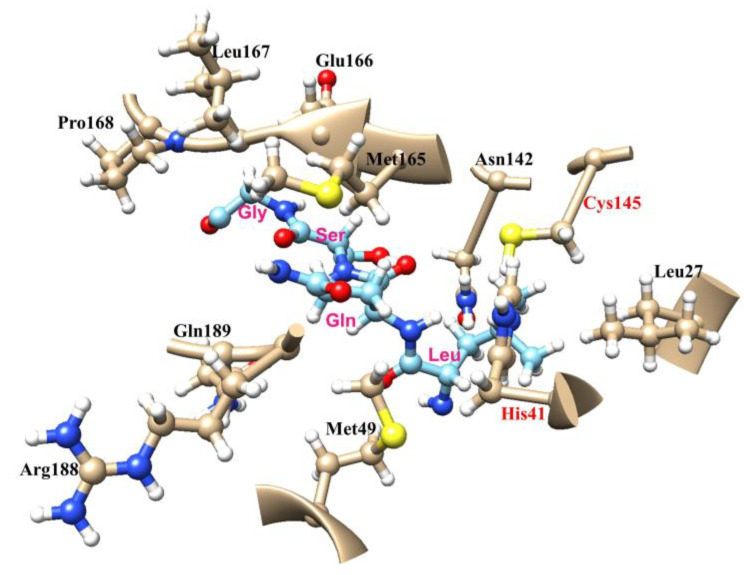
The interactions between M^pro^_SARS2_ and key amino acids (LQSG) in the substrate are predicted by molecular docking.

Here, we carried out molecular docking to gain a better understanding of the interaction between M^pro^ and its substrate. The docking results revealed that the substrates bind to M^pro^_SARS2_ at both the active site (Binding Site 1) and the surface (Binding Site 2) of M^pro^_SARS2_, with a distribution of 65% and 35%, respectively (Figure S4 and Table S2). The average effective binding energy (ΔG*) at the active site and surface was −6.4 kcal/mol and −3.6 kcal/mol, respectively, compared to the ΔG* values of M^pro^_SARS_ of −5.8 kcal/mol (active site) and −3.7 kcal/mol (surface) (Tables S2 and S3). Notably, the average effective binding energy at the active site of M^pro^_SARS_ was lower than that of M^pro^_SARS2_, which explains why the enzyme catalytic efficiency of M^pro^_SARS2_ was slightly higher than that of M^pro^_SARS_. Figure 3 shows the interactions between M^pro^_SARS2_ and key amino acids (LQSG) in the substrate (Figure S5 for M^pro^_SARS_). Hydrophobic interactions between M^pro^ and substrate involved Met49, Met165, Arg188, and Glu189. The side chains of Met165 and Leu167 played important roles in substrate recognition, while Glu166 contributed to the stabilization of the substrate [23].

To obtain more information about M^pro^ and explore why mutating the active sites would cause lower enzyme activity, the molecular docking of H41A and C145A was measured. The docking results showed that the possibility of substrate binding to the active site of the protease was reduced from 65% for WT to around 19% for both H41A and C145A mutants. The average effective binding energy ΔG* at the active site increased from −6.4 kcal/mol (WT) to −1.8 kcal/mol (H41A) and −1.9 kcal/mol (C145A) (Table S2), indicating that the substrate’s binding affinity weakens upon mutation compared to WT. There was a similar trend observed in SARS-CoV (Table S3). As is widely known, M^pro^ harbors a catalytic dyad comprised of His41 and Cys145 in the active site, which is formed by four major pockets (S1: Phe140, Leu141, Asn142, Ser144, His163, Met165, Glu166, His172, and Ser1 from a neighboring protomer; S2: His41, Met49, Tyr54, M165, and Asp187; S1′: Thr25, Thr26, Leu27, His41, and C145; S4: Met165, Leu167, Phe185, Gln192, and Gln189). The S1 pocket is the most conserved subsite and is only occupied by glutamine, which is the most important residue among all eleven cleavage sites of M^pro^ [23]. Furthermore, at the center of the active site, the complete carboxyl terminus of glutamine at position 1 (P1 site) is close to the thiol group of the Cys145 nucleophile, whose thiol sulfur is 3.8 Å from the Nε2 of the base H41 [23]. His41 and Cys145 are critical in forming the S1′ and S2 substrate pockets, which impact substrate binding. Furthermore, as one of the most important amino acids in the substrate, the main chain carbonyl oxygen of glutamine occupies the oxyanion hole, which is stabilized by the amide groups of Gly143 and Cys145. The Cys145 residue also interacts with the side chain of serine in the substrate through van der Waals interactions and can form a covalent bond with some inhibitors, such as N3 and 11a [23]. Therefore, once the active site at position 41 or 145 is mutated, the optimal binding mode between substrate and protease will change, ultimately affecting the binding efficiency of the substrate. To further verify the results, the molecular docking of M^pro^ with Calpeptin, a substance with higher inhibition of M^pro^_SARS2_, was carried out. The percentage of Calpeptin binding at the active site by docking was decreased from 60% to 41% and 47% with increasing average binding energy ΔG* from −4.5 kcal/mol to −3.0 kcal/mol, and −3.6 kcal/mol for WT, H41A, and C145A, respectively (Table S2). All these results suggested that the interaction of the substrate and protein will be dismissed upon mutation at the position of either His41 or Cys145.

### 2.4. Comprehensive Insights into the Enzyme Activity

The accurate characterization of M^pro^, including *k*_cat_ and K_m_, is critical in selecting inhibitors and determining substrate specificity [23,56]. However, the kinetic parameters of M^pro^ can vary significantly among different studies measured by different methods, even those utilizing the same method [25,41,42]. The catalytic efficiency of M^pro^ measured by the FRET-based cleavage assay was 26500 ± 1131 M^−1^s^−1^, while it was measured at 2348 ± 636 M^−1^s^−1^ by the LC-MS method [25,35]. Thus, it is essential to have a clear view of the kinetic behaviors of M^pro^. In our study, the kinetic behaviors of M^pro^ from both SARS-CoV-2 and SARS-CoV were measured by using both the FRET-based cleavage assay and the LC-MS method. The K_m_ value, measured by the FRET-based cleavage assay, was at the micromolar level, while it was at the millimolar level when using the LC-MS method. The *k*_cat_/K_m_ value measured by the FRET-based cleavage assay was higher than that of LC-MS. Combined with all kinetic parameters of M^pro^ measured by FRET-based cleavage assay, we found that for M^pro^_SARS2_, the *k*_cat_ value ranges from 0.01 to 0.26 s^−1^. The *k*_cat_ measured in our study is 0.23 ± 0.01 s^−1^, which is comparable to 0.21 ± 0.01 s^−1^ (Wang group’s data) [43]. The reported K_m_ values range from 11 to 228 μM, while the K_m_ value in our study (34.2 ± 4.8 μΜ) falls within this range [17,32,43]. There are not much data from previous studies regarding the kinetic parameters of M^pro^_SARS2_ using the LC-MS method, most probably due to its expensive cost and time-consuming nature.

For M^pro^_SARS_, the *k*_cat_ value ranges from 0.005 to 1.9 s^−1^ (in this study, we reported a *k*_cat_ value of 0.17 ± 0.01 s^−1^). The K_m_ value measured in our study is 61.5 ± 6.1 μΜ, while this value ranges from 9 to 890 μΜ in other studies. The catalytic efficiency (*k*_cat_/K_m_) exhibited a wide range from 3011 to 111,765 M^−1^s^−1^ [25,35,41,45,46]. When using the LC-MS method to measure the kinetic parameters, the *k*_cat_ value ranged from 0.2 to 6.4 s^−1^, with the K_m_ value ranging from 230 to 2638 μΜ. The calculated *k*_cat_/K_m_ values showed a broad range from 35.5 to 2441 M^−1^s^−1^. The observed discrepancy may be due to variations in fluorophores present in the substrates, differences in methodologies employed, or varying states of the target protein in different studies.

From the gel filtration analysis and the catalytic efficiency between the authentic M^pro^ (in dimer form) and tagM^pro^ (mostly in the monomeric state), the catalytic efficiency was much lower compared with the authentic M^pro^, suggesting the tag does have an influence on dimerization and further affects the enzyme activity of the M^pro^. Furthermore, the absence of the N-terminal was also observed to affect the aggregation of the M^pro^. Previous studies have shown that the 1–4 amino acids truncated protease at the N-terminal results in little enzyme activity, mainly in monomer form. However, 1–3 amino acids truncated protease maintains the dimeric state and retains the enzyme activity [21]. These results indicated that there was a direct relationship between the catalytic mechanism and the quaternary structure of M^pro^. The catalytic machinery consists of the catalytic dyad His41-Cys145, oxyanion-loop Phe140-Cys145, and those residues critical for the binding substrate. Previous studies have shown that mutating Arg298 to alanine leads to irreversible inactivation and monomer formation in M^pro^ [59]. In comparison, the N214A mutation inactivated the enzyme without significant structure changes [60]. In our study, we mutated the catalytic dyad to alanine and observed a significant reduction in enzyme activity in these mutants (H41A and C145A). Molecular docking results showed that the catalytic efficiency of M^pro^_SARS2_ was higher than that of M^pro^_SARS_. Furthermore, the active site mutants (H41A and C145A) were constructed, and the kinetic parameters were further measured to assist us in gaining a compressive understanding of the reduced enzyme efficiency of mutants compared with the wild type at the atomic level.

## 3. Conclusions

As there are substrates with different fluorophores, different measurement methods, different forms of M^pro^, and even different assay conditions among various groups, the kinetic behaviors of the M^pro^ are confusing. Therefore, it is necessary to make a clear view. In our study, we used the FRET-based cleavage assay and LC-MS method to determine the catalytic efficiency of M^pro^ (with or without tag), and compared these results with almost all the previous research’s kinetic parameters of the target protein. Our goal was to find a suitable principle to determine the kinetic behaviors of M^pro^.

One important conclusion we made is that the catalytic efficiency of M^pro^ measured by the FRET-based cleavage assay was higher than that measured by using the LC-MS method. Although the LC-MS method was considered a more reliable way to obtain kinetic parameters, it was more costly and time-consuming. Both methods have their limitations and advantages, and we hope that future research can develop a more effective and accurate technique to determine the kinetic behaviors of M^pro^ based on our findings.

Furthermore, we found that the tag on the protein would affect the dimerization and catalytic efficiency of M^pro^. Comparing the enzyme activity between wild-type and mutants, we obtained much more detailed information about the kinetic behaviors of M^pro^. As accurately selective inhibitors against M^pro^_SARS2_ could promote the discovery of potential broad-spectrum drug candidates to fight against CoV infectious, our comprehensive study of M^pro^ kinetic behaviors provides valuable insights for inhibitor selection.

## 4. Materials and Methods

### 4.1. Cloning, Protein Expression, and Purification of M^pro^

The coding sequences of M^pro^ from SARS-CoV-2 (NC_045512) and SARS-CoV (NC_004718) were synthesized by the GENEWIZ Company (Tianjin, China). To obtain the tag M^pro^, the SARS-CoV-2 M^pro^ sequence was inserted into the NheⅠ and XhoⅠ sites of pET21a plasmid (tag_MAS_M^pro^_LE6×His_). The SARS-CoV M^pro^ sequence was inserted into the BamHⅠ and HindⅢ sites of pET28a plasmid (tag_34aa_M^pro^) (Figure S6a).

These target plasmids were transformed into *Escherichia coli* BL21(DE3) cells. Cultures were grown at 37 °C in 1 L LB medium containing corresponding antibiotic (100 μg/mL ampicillin or 50 μg/mL kanamycin) until the OD_600_ reached 0.8 and then was induced with 0.5 mM isopropyl-1-thio-β-galactopyranoside (IPTG) at 16 °C for 16 h. Wet cells were obtained by centrifuging at 5000× *g* for 20 min. The purification processes were taken at 4 °C or ice unless specified. The collected wet cell was resuspended by the lysis buffer (40 mM Tris-HCl, 100 mM NaCl, 10 mM imidazole, and 7.5 mM β-mercaptoethanol, pH 8.0) before ultrasonic, lysozyme, DNAase, and phenylmethanesulfonyl fluoride (PMSF) were added to the buffer. After ultrasonication, the supernatant was obtained by centrifuging at 5000× *g* for 20 min, followed by filtration with the 0.45 μm filter. The target protein was then purified using a pre-equilibrated Ni-NTA metal affinity column with lysis buffer. The column was washed with the lysis buffer for 10 column volumes. Additionally, the elution buffer (40 mM Na_2_HPO_4_-NaH_2_PO_4_, 100 mM NaCl, 250 mM imidazole, and 7.5 mM β-mercaptoethanol, pH 7.4) was used to elute the target protein. Finally, the target protein was concentrated by storage buffer (40 mM Na_2_HPO_4_-NaH_2_PO_4_, 100 mM NaCl, and 1 mM DTT, pH 7.4) and stored at −80 °C for further use. The concentration of the target protein was measured by Bradford assay.

To obtain the authentic target protein (native M^pro^), the pGSTM expression system was constructed (Figure S6b) [35], and the DNA sequence of M^pro^ (SARS-CoV-2 or SARS-CoV) was inserted into BamH1 and Xho1 sites of pGEX-4T-1 plasmid with GST (Glutathione S-transferase with 218 amino acids) tag at the N-terminal and a 6×His at the C-terminus. At the N terminus, five amino acids (SAVLQ) corresponding to the P5-P1 sites of the N-terminal autocleavage sequence of M^pro^ were introduced between the GST tag and the first residue of the protease. Thus, the authentic N terminus would become available by autocleavage during protein expression for WT. At the C terminus, eight amino acids GPHHHHHH (GPH6) were added after the last glutamine residue. Because the GP corresponded to the P1′ and P2′ sites, the last two amino acids at the C-terminal of M^pro^ corresponded to the P2 and P1 sites of the rhinovirus 3C protease. An authentic C terminus could be obtained following cleavage by rhinovirus 3C protease through the strategy.

For pGSTM expression system, after the target protein was eluted, the GP6×His tag was cleaved by rhinovirus 3C protease at 4 °C overnight and then purified by GST affinity resin and Ni-NTA metal affinity column. The reaction buffer for rhinovirus 3C protease contained 50 mM sodium phosphate, 150 mM NaCl, 1 mM EDTA, and 1 mM DTT (pH 8.0). The resulting protein was further purified by anion-exchange chromatography using a HiTrap Q column in a linear gradient from 25 mM to 500 mM NaCl with 20 mM Tris-HCl, 10% glycerol, and 1 mM DTT (pH 8.0). The purified proteins were analyzed by SDS-PAGE, as shown in Figure S1.

Based on the pGSTM system, H41A and C145A mutagenesis (Figure S6c) were obtained using a Fast Site-Directed Mutagenesis kit (TIANGEN, Beijing, China) with the primers shown in Table S1. Due to the low enzyme activity of mutants at H41A and C145A, the GST tag cannot be cleaved during expression, resulting in the tag protein tag_GST_H41A_GP6×His_ (61.4 kDa) and tag_GST_C145A_GP6×His_ (61.4 kDa) with both GST at N-terminal and eight amino acids GPHHHHHH (GPH6) at C-terminal (Figure S1). The authentic forms of H41A and C145A were further obtained by using authentic M^pro^ (without any tag) to cleave the GST tag at 4 °C for 4 h. The mutated protein with only the GPH6 tag at the C-terminal was then purified using a Ni-NTA metal affinity column. Finally, the GPH6 tag was cleaved using the rhinovirus 3C enzyme, and the resulting mutant proteins without any tag were further purified as WT mentioned above (Figure S1). To obtain an overall understanding of the kinetic behaviors of M^pro^, the catalytic efficiency of tag_GST_H41A_GP6×His_, tag_GST_C145A_GP6×His_, H41A, and C145A were also measured (Table 1, lines 12 to 15 for SARS-CoV-2; Table 2, lines 17 to 20 for SARS-CoV).

### 4.2. Analytical Gel Filtration

The aggregation state of M^pro^ and tagM^pro^ (SARS-CoV-2 and SARS-CoV) were analyzed using Uniondex 75pg 16/60 column (Union-Biotech, Shanghai, China). Different concentrations of proteins with 0.5 mL 4 mg/mL and 2 mL 0.2 mg/mL were injected into the column and eluted with the 40 mM Tris-HCl, 100 mM NaCl, 1 mM DTT (pH 8.0) buffer at a flow rate of 0.5 mL/min, monitored at 280 nm on fast protein liquid chromatography (Figure 1). The result was calibrated using the SEC standard curve (Figure S7).

### 4.3. Enzyme Activity Assay and Kinetic Parameters Measurement

The substrate NH_2_–Thr–Ser–Ala–Val–Leu–Gln–Ser–Gly–Phe–Arg–COOH was synthesized by the GenScript Company (Nanjing, China). Cleavage reactions were incubated at 30 °C and contained 2 μM M^pro^, 0.5 mM substrate, 20 mM Na_2_HPO_4_, 200 mM NaCl, 1 mM EDTA, and 1 mM DTT (pH 7.6) in a total volume of 60 μL. The reaction was quenched using an equal volume of 2% formic acid. The cleavage products were resolved by HPLC (Agilent 1260, Beijing, China) using C18 reversed-phase chromatographic column (4.6 × 150 mm, Agilent, Beijing, China) with a 15 min linear gradient of 5–60% acetonitrile at 1 mL/min flow rate. The absorbance was monitored at 215 nm and 260 nm. To determine the kinetic parameters of M^pro^, 50 nM WT enzyme (15 to 30 μM for mutants) was incubated with the substrate (0.25–4 mM) in reaction buffer (20 mM Na_2_HPO_4_, 200 mM NaCl, 1 mM EDTA, and 1 mM DTT, pH 7.6). The reaction was quenched using 2% formic acid every 2 min (t = 2 min, 4 min, 6 min, and 8 min). The identities of products were confirmed by mass spectrometry (Agilent Technologies 6420 Triple Quad LC/MS, Beijing, China). The multiple reaction monitoring method was used to detect the content of the product. The precursor → product transition detected by MS/MS system is *m*/*z* 616.3→144.9 for the product (TSAVLQ) (Figures S8–S10). The peak areas were integrated to quantify the product. The standard curve was obtained by measuring varied concentrations (7, 15, 31, 62, 125, 250, and 500 μM) of the product (TSAVLQ). The corresponding product was calculated by fitting the standard curve and then obtained the velocity under various concentrations of substrate. The Michaelis–Menten plot was treated by GraphPad Prism software (GraphPad Prism 5).

The fluorescent substrate Dabcyl-KTSAVLQSGFRKM-E(Edans)-NH_2_ was synthesized by the GenScript Company (Nanjing, China). The reaction buffer was 20 mM Tris (pH 7.6), 100 mM NaCl, 1 mM EDTA, and 1 mM DTT. In the fluorescence resonance energy transfer (FRET)-based cleavage assay, the Edans group was released once the M^pro^ cleaved the substrate. The fluorescence signal of Edans was detected at an emission wavelength of 460 nm with excitation at 360 nm using Infinite 200 PRO fluorescence spectrometry (TECAN, Switzerland). Firstly, 2 μL enzyme (50 nM for WT and 2 to 100 μM for mutants) was added into a 96-well plate containing 48 μL reaction buffer. The reaction was initiated by adding 50 μL substrate, which was dissolved in different concentrations (5, 10, 20, 40, 80, 100, 120, 180, 200, 250, and 300 μM). The fluorescence value was read by Infinite 200 PRO fluorescence spectrometry every 1 min. The standard curve was obtained by measurement of the varied concentrations of free Edans (from 0.5 to 20 μM). The linear section of the curve was used to calculate the initial velocities. The corresponding relative fluorescence intensity per minute was converted to the cleavage substrate product by fitting it to the calibration curve of free Edans.

### 4.4. Molecular Docking

The process of molecular docking was accomplished by the SwissDock website (www.swissdock.ch/docking, accessed on 17 January 2022) [61]. The structure of target protein M^pro^ (SARS-CoV-2, PDB:6M03; SARS-CoV, PDB: 1JU1) and the substrate (PDB: 2Q6G) was taken from the PDB website (www.rcsb.org, accessed on 15 January 2022). The structure of Calpeptin was optimized by Gaussian 09 package with B3LYP/def2TZVP basis set. The structure of H41A and C145A were constructed by Pymol 2.3 (the structure templates were 6M03 and 1JU1). The predicted binding modes were visualized and further analyzed by UCSF Chimera 1.16 package. To enhance the reliability of the results, we conducted further analysis of the data. Firstly, we identified all possible binding modes by performing molecular docking, which included substrate binding near the active site (marked AS) and on the surface (marked SF) (Figure S4). We defined the proportion of binding modes near the active site out of all possible binding modes as the effective binding ratio (BS%), which accurately reflects the substrate’s tendency to bind to the active site (Tables S2 and S3). Secondly, we introduced the concept of average binding energy, which represents the average value of binding energy (ΔG*) calculated by the molecular docking method in the AS or SF area. Lastly, we defined the product of BS% and the corresponding average binding energy as the effective average binding energy, which can be used to reflect the potential affinity of the target protein and substrate.

## Figures and Tables

**Table 1:** Comparison of the M^pro^ kinetic parameters using different methods from SARS-CoV-2 *^a^*.

#	Method	Enzyme *^b^*	*k*_cat_ (s^−1^)	K_m_ (μM)	*k*_cat_/K_m_ (M^−1^s^−1^) *^c^*	Substrate	Ref.
1	FRET	M^pro^			28,500	MCA–AVLQSGFRK(Dnp)K	[32]
2	FRET	tagM^pro^_LE6×his_	0.21 ± 0.01	30.9 ± 3.8	6689 ± 885	Dabcyl-KTSAVLQSGFRKME(Edans)	[43]
3	FRET	M^pro^	0.16 ± 0.01	27.8 ± 5.2	5748 ± 1135	Dabcyl-KTSAVLQSGFRKME(Edans)	[43]
4	FRET	tagM^pro^_6×his_	0.04	11	3640	Dabcyl-KTSAVLQSGFRKME(Edans)	[44]
5	FRET	M^pro^			3426.1 ± 416.9	Dabcyl-KTSAVLQSGFRKM-E(Edans)	[45]
6	FRET	M^pro^	0.18 ± 0.02	207.3 ± 12	859 ± 57	Ac-Abu-Tle-LQ-ACC	[17]
7	FRET	M^pro^	0.14 ± 0.006	189.5 ± 2.7	760 ± 50	Ac-Thz-Tle-LQ-ACC	[17]
8	FRET	M^pro^	0.05 ± 0.002	228.4 ± 9.9	219 ± 3	Ac-Val-Lys-LQ-ACC	[17]
9	FRET	tag_HM_M^pro^	0.01 ± 0.0009	53.1 ± 8.1	214 ± 36	Dabcyl-KTSAVLQSGFRKME(Edans)	[43]
10	FRET	M^pro^	0.23 ± 0.01	34.2 ± 4.8	6800 ± 976	Dabcyl-KTSAVLQSFRKME(Edans)	[Figure 2]
11	FRET	tag_MAS_M^pro^_LE6×His_	0.9 ± 0.1 × 10^−2^	139 ± 22.2	67.5 ± 11.8		[Figure 2]
12	FRET	C145A	1.5 ± 0.1 × 10^−2^	47.0 ± 7.7	319.3 ± 54.7		[Figure S2]
13	FRET	H41A	2.2 ± 0.1 × 10^−2^	74.7 ± 13.4	292.1 ± 55.5		[Figure S2]
14	FRET	tag_GST_C145A_GP6×His_	3.5 ± 0.2 × 10^−3^	48.5 ± 8.9	71.8 ± 13.7		[Figure S2]
15	FRET	tag_GST_H41A_GP6×His_	2.4 ± 0.1 × 10^−5^	20.9 ± 3.7	1.1 ± 0.2		[Figure S2]
16	LC-MS	M^pro^	2.2 ± 0.07	903.5 ± 86.9	2444 ± 248	NH2-TSAVLQSGFR-COOH	[Figure 2]
17	LC-MS	tag_GST_C145A_GP6×His_	9.6 ± 1.0 × 10^−5^	1306 ± 316.2	7.4 ± 1.9 × 10^−2^		[Figure S3]
18	LC-MS	tag_GST_H41A_GP6×His_	1.1 ± 0.06 × 10^−4^	570.9 ± 110.2	20.1 ± 4.1 × 10^−2^		[Figure S3]

*^a^* To facilitate the comparison, all units were converted to s^−1^, μM, or M^−1^s^−1^. *^b^* Abbreviations: M^pro^ = protein without tag; tagM^pro^_LE6×his_ = C terminal with Leu-Glu-6×His; tagM^pro^_6×his_ = C terminal with 6×His; tag_HM_M^pro^ = N terminal with His-Met; tag_MAS_M^pro^_LE6×his_ = N terminal with Met-Ala-Ser and C terminal with Leu-Glu-6×His; tag_GST_C145A_GP6×His_ = N terminal with GST protein and C-terminal with GPHHHHHH; and tag_GST_H41A_GP6×His_ = N terminal with GST protein and C-terminal with GPHHHHHH. *^c^* The order of the table is based on the *k*_cat_/K_m_ value from previous studies or in our study using FRET-based cleavage assay and LC-MS method.

**Table 2:** Comparison of the M^pro^ kinetic parameters using different methods from SARS-CoV *^a^*.

#	Method	Enzyme *^b^*	*k*_cat_ (s^−1^)	K_m_ (μM)	*k*_cat_/K_m_ (M^−1^s^−1^) *^c^*	Substrate	Ref.
1	FRET	M^pro^	1.9 ± 0.1	17 ± 4	111,765 ± 26,947	Dabcyl-KTSAVLQSGFRKME-Edans	[41]
2	FRET	M^pro^			29,000	Dabcyl-SAVLQSGFRK- Edans	[25]
3	FRET	M^pro^	1.06 ± 0.04	40 ± 0.8	26,500 ± 1131	MCA-AVLQSGFR-Lys(Dnp)-Lys-NH2	[35]
4	FRET	tagM^pro^_GP-6×his_	0.41 ± 0.02	61 ± 2.9	6800 ± 457	MCA-AVLQSGFR-Lys(Dnp)-Lys-NH2	[35]
5	FRET	tag_NHPFT_M^pro^	0.03 ± 0.0001	9 ± 1	3667 ± 407	Dabcyl-LAQAVRSSSR-Edans	[46]
6	FRET	M^pro^			3011.3 ± 294.6	Dabcyl-KTSAVLQSGFRKM-E(Edans)	[45]
7	FRET	tag_GS_M^pro^	0.14 ± 0.01	129 ± 7	1085 ± 97	MCA-AVLQSGFR-Lys(Dnp)-Lys-NH2	[47]
8	FRET	1–192 aa	0.01 ± 0.001	13 ± 2	1077 ± 182	Dabcyl-LAQAVRSSSR-Edans	[46]
9	FRET	tag_GPLGS_M^pro^	0.02 ± 0.001	126 ± 8	167 ± 13	MCA-AVLQSGFR-Lys(Dnp)-Lys-NH2	[35]
10	FRET	tagM^pro^_6×his_	0.07 ± 0.03	850 ± 410	86 ± 54	TSAVLQ-pNA	[48]
11	FRET	tag_28aa-6×his_M^pro^	0.01	145.69	69.05	Abz-TSAVLQSGFRK-DNP	[49]
12	FRET	tag_4aa-6×his-2aa_M^pro^	0.02	404	45	Edans-VNSTLQSGLRK(Dabcyl)-M	[50]
13	FRET	tag_28aa-6×his_M^pro^	0.005	252.5	20	Abz-SGVTFQGKFKK-DNP	[49]
14	FRET	tagC145S_6×his_	0.004 ± 0.001	890 ± 270	4.3 ± 1.7	TSAVLQ-pNA	[48]
15	FRET	M^pro^	0.17 ± 0.01	61.5 ± 6.1	2792 ± 293		[Figure 2]
16	FRET	tag_34aa_M^pro^	0.9 ± 0.04 × 10^−3^	50.8 ± 9.9	17.0 ± 3.5	Dabcyl-KTSAVLQSGFRKME(Edans)	[Figure 2]
17	FRET	C145A	2.6 ± 0.1 × 10^−3^	50.5 ± 6.5	51.3 ± 6.9		[Figure S2]
18	FRET	H41A	1.5 ± 0.1 × 10^−3^	83.2 ± 15.5	18.5 ± 3.7		[Figure S2]
19	FRET	tag_GST_C145A_GP6×His_	6.8 ± 0.2 × 10^−5^	8.4 ± 1.4	8.0 ± 1.3		[Figure S2]
20	FRET	tag_GST_H41A_GP6×His_	2.1 ± 0.1 × 10^−5^	10.2 ± 2.4	2.0 ± 0.5		[Figure S2]
21	LC-MS	M^pro^	0.54 ± 0.04	230 ± 60	2348 ± 636	NH2-TSAVLQSGFRKW-COOH	[25]
22	LC-MS	tag_GS_M^pro^			1032	NH2-SWTSAVLQSGFRKWA-COOH	[51]
23	LC-MS	tagM^pro^_6×his_	0.20 ± 0.05	1150 ± 280	177 ± 60	NH2-TSAVLQSGFRK-COOH	[42]
24	LC-MS	tagM^pro^_6×his_ *^d^*			131	NH2-SAVLQSGF-COOH	[26]
25	LC-MS	tagM^pro^_6×his_ *^e^*			35.5	NH2-SAVLQSGF-COOH	[26]
26	LC-MS	tagC145S_6×his_			0.09	NH2-TSAVLQSGFRK-COOH	[48]
27	LC-MS	tagC145S_6×his_			0.01	NH2-ATVRLQAGNAT-COOH	[48]
28	LC-MS	M^pro^	6.4 ± 0.8	2638 ± 638.1	2441 ± 662	NH2-TSAVLQSGFR-COOH	[Figure 2]
29	LC-MS	tag_GST_C145A_GP6×His_	7.6 ± 1.2 × 10^−4^	859.8 ± 331.4	0.9 ± 0.4		[Figure S3]
30	LC-MS	tag_GST_H41A_GP6×His_	2.2 ± 0.1 × 10^−5^	525.1 ± 135.5	4.2 ± 1.1 × 10^−2^		[Figure S3]

*^a^* To facilitate the comparison, all units were converted to s^−1^, μM, or M^−1^s^−1^. *^b^* Abbreviations: tagM^pro^_GP-6×his_ = C terminal with Gly-Pro-6×His; tag_NHPFT_M^pro^ = N terminal with Asn-His-Pro-Phe-Thr; tag_GS_M^pro^ = N terminal with Gly-Ser; 1–192 aa = the protein was expressed from 1–199 aa; tag_GPLGS_M^pro^ = N terminal with Gly-Pro-Leu-Gly-Ser; tagM^pro^_6×his_ = C terminal with 6×His; tag_28aa-6×his_M^pro^ = N terminal with 28 extra amino acids and 6×His; tag_4aa-6×his-2aa_M^pro^ = N terminal with Met-Arg-Gly-Ser-6×His-Gly-Ser; tag_28aa-6×his_M^pro^ = N terminal with 28 extra amino acids and 6×His; tagC145S_6×his_ = C terminal with 6×His; tag_34aa_M^pro^ = N terminal with 34 extra amino acids; tag_GST_C145A_GP6×His_ = N terminal with GST protein and C-terminal with GPHHHHHH; a nd tag_GST_H41A_GP6×His_ = N terminal with GST protein and C-terminal with GPHHHHHH. *^c^* The order of the table is based on the *k*_cat_/K_m_ value from previous studies or in our study using FRET-based cleavage assay and LC-MS method. *^d^* The concentration of the enzyme is 7.41 μM.; *^e^* the concentration of the enzyme is 5.14 μM.

## Data Availability

Data are contained within the article or supplementary material.

## Supplementary Materials

The following supporting information can be downloaded at https://www.mdpi.com/article/10.3390/molecules28124605/s1.
