# Supplementary material for: Comprehensive Understanding of the Kinetic Behaviors of Main Protease from SARS-CoV-2 and SARS-CoV: New Data and Comparison to Published Parameters
Source: Molecules. 2023 Jun 7;28(12):4605. doi: 10.3390/molecules28124605 (PMC10304930; doi:10.3390/molecules28124605)
Supplement: Supplementary file 1 [file molecules-28-04605-s001.zip › molecules-2364257-supplementary.pdf]

<u>Supplementary Information</u>

Comprehensive Understanding of the Kinetic Behaviors of Main Protease from SARS-CoV-2 and SARS-CoV: New Data and Comparison to Published Parameters

Fangya Li, Tingting Fang, Feng Guo, Zipeng Zhao, and Jianyu Zhang*

School of Pharmaceutical Science and Technology, Tianjin University, Tianjin 300072, China

*Correspondence address. Tel/Fax: +086-22-8740-1830; E-mail: Jianyu.Zhang@tju.edu.cn

Table S1. Primers used in this work

Primers	Sequence(5’-3’)
**H41A$_{SARS2}$-F**	GGTGTATTGCCCAAGAGCCGTGATTTGCACTAGTGAAG
**H41A$_{SARS2}$-R**	CACGGCTCTTGGGCAATACACCACATCATCCAGCCATAGG
**C145A$_{SARS2}$-F**	GCTTTCTGAATGGCAGCGCCGGCAGTGTGGGCTTTAAC
**C145A$_{SARS2}$-R**	CTGCCGGCGCTGCCATTCAGAAAGCTGCCTTTAATGG
**H41A$_{SARS}$-F**	CGTGTATTGCCCGCGCGCGGTGATTTGCACCGCGGAAG
**H41A$_{SARS}$-R**	CAAATCACCGCGCGCGGGCAATACACGGTATCATCCAGC
**C145A$_{SARS}$-F**	GCTTTCTGAACGGCAGCGCCGGCAGCGTGGGCTTTAAC
**C145A$_{SARS}$-R**	GTTAAAGCCCACGCTGCCGGCGCTGCCGTTCAGAAAGC

Table S2. Swiss Docking about M^{pro} with the substrate (TSAVLQSGFR) and calpeptin from SARS-CoV-2

M^{pro}	Average binding energyΔG* (kcal/mol)[a]		Effective binding ratio (BS%)[b]		Average effective binding energyΔG* (kcal/mol)[c]	
	Active site	Surface	Active site	Surface	Active site	Surface
**With substrate**						
**WT**	-9.8	-10.4	0.65	0.35	-6.4	-3.6
**H41A**	-9.6	-9.6	0.19	0.81	-1.8	-7.8
**C145A**	-10.4	-9.7	0.19	0.81	-1.9	-7.9
**With calpeptin**						
**WT**	-7.4	-7.2	0.60	0.40	-4.5	-2.9
**H41A**	-7.5	-7.3	0.41	0.59	-3.0	-4.3
**C145A**	-7.6	-7.3	0.47	0.53	-3.6	-3.8

[a] The average binding energyΔG* was calculated by molecular docking method in the active site or surface area. [b] The proportion of binding modes near the active site out of all possible binding modes is defined as the effective binding ratio (BS%). [c] The product of BS% and corresponding average binding energy was defined as effective average binding energy.

Table S3. Swiss Docking about M^{pro} with the substrate (TSAVLQSGFR) and calpeptin from SARS-CoV

**M^{pro}**	**Average binding energyΔG* (kcal/mol)**[a]		**Effective binding ratio (BS%)**[b]		**Average effective binding energyΔG* (kcal/mol)**[c]	
	Active site	Surface	Active site (SA)	Surface (SF)	Active site	Surface
			**With substrate**			
**WT**	-9.7	-9.2	0.60	0.40	-5.8	-3.7
**H41A**	-9.4	-9.2	0.50	0.50	-4.7	-4.6
**C145A**	-9.5	-9.0	0.50	0.50	-4.8	-4.5
			**With calpeptin**			
**WT**	-7.2	-7.2	0.66	0.34	-4.7	-2.5
**H41A**	-7.3	-7.1	0.63	0.37	-4.6	-2.7
**C145A**	-7.3	-7.2	0.58	0.42	-4.2	-3.1

[a] The average binding energyΔG* was calculated by molecular docking method in the active site or surface area. [b] The proportion of binding modes near the active site out of all possible binding modes is defined as the effective binding ratio (BS%). [c] The product of BS% and corresponding average binding energy was defined as effective average binding energy.

(a)

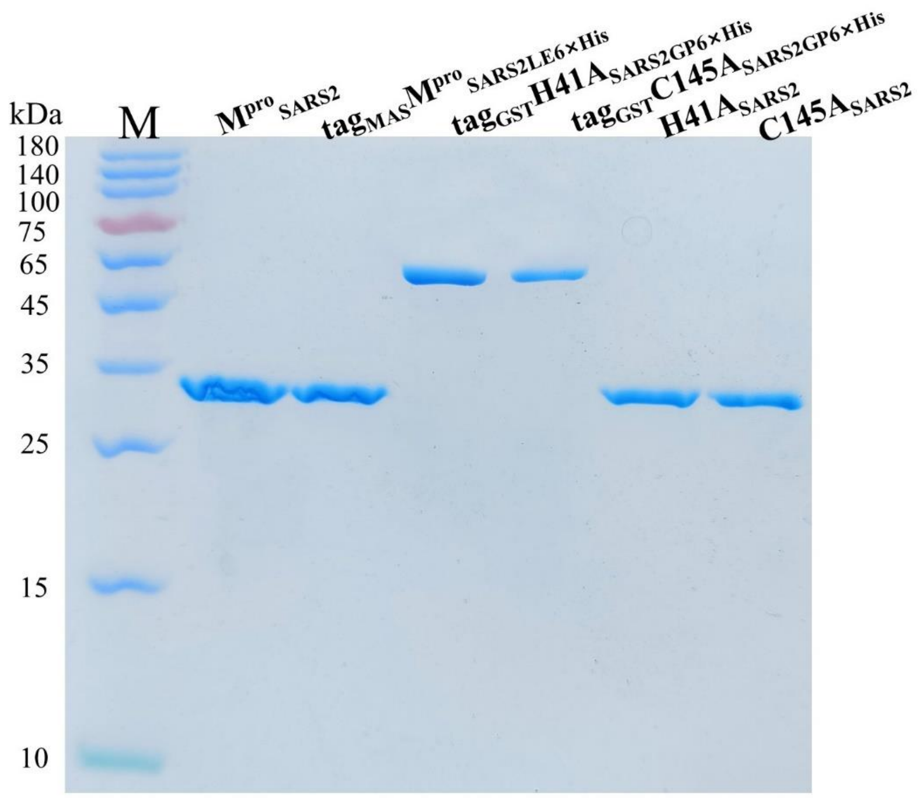

(b)

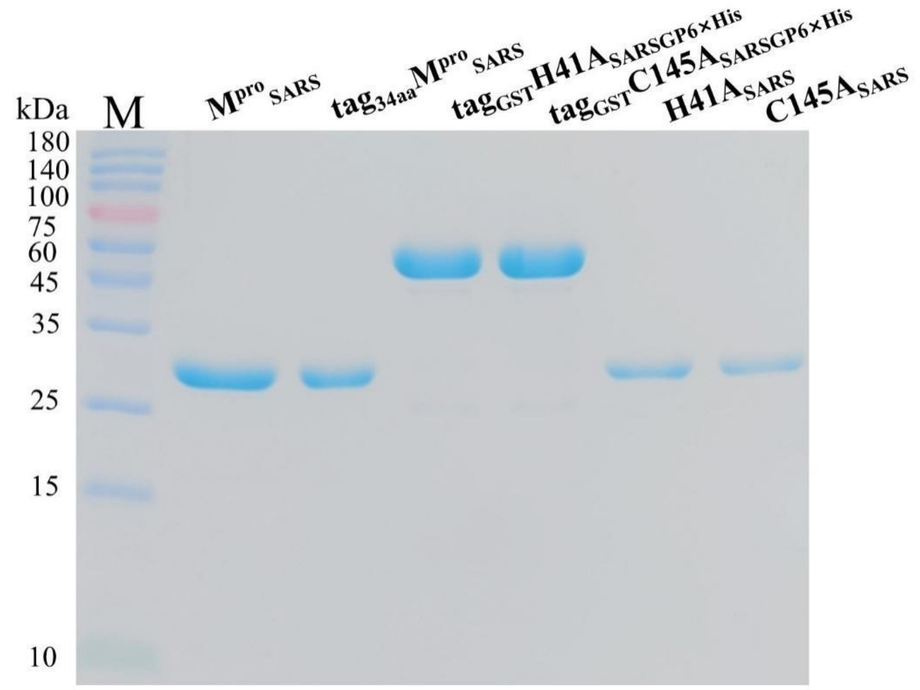

Figure S1. The SDS-PAGE of the main protease (M^{pro}) and mutants. (a) M, molecular weight, line 1, M$^{pro}{}_{SARS2}$, line 2, tag$_{MAS}$M$^{pro}{}_{SARS2LE6\times His}$ = N terminal with Met-Ala-Ser and C terminal with Leu-Glu-6×His, line 3, tag$_{GST}$H41A$_{SARS2GP6\times His}$ = N terminal with

GST protein (218 amino acids, around 26.6 kDa) plus C- terminal GPH6 (8 amino acids, around 1 kDa), line 4, $tag_{GST}C145A_{SARS2GP6\times His}$ = N terminal with GST protein (218 amino acids, around 26.6 kDa) plus C- terminal GPH6 (8 amino acids, around 1 kDa), line 5, $H41A_{SARS2}$, line 6, $C145A_{SARS2}$. (b) M, molecular weight, line 1, M^{pro}_{SARS}, line 2, $tag_{34aa}M^{pro}_{SARS}$ =N terminal with 34 extra amino acids, line 3, $tag_{GST}H41A_{SARSGP6\times His}$= N terminal with GST protein (218 amino acids, around 26.6 kDa) plus C- terminal GPH6 (8 amino acids, around 1 kDa), line 4, $tag_{GST}C145A_{SARSGP6\times His}$= N terminal with GST protein (218 amino acids, around 26.6 kDa) plus C- terminal GPH6 (8 amino acids, around 1 kDa), line 5, $H41A_{SARS}$, line 6, $C145A_{SARS}$. The size of $tag_{GST}H41A_{SARS2GP6\times His}$ ($tag_{GST}C145A_{SARS2GP6\times His}$, $tag_{GST}H41A_{SARSGP6\times His}$, and $tag_{GST}C145A_{SARSGP6\times His}$) is around 61.4 kDa

(a)

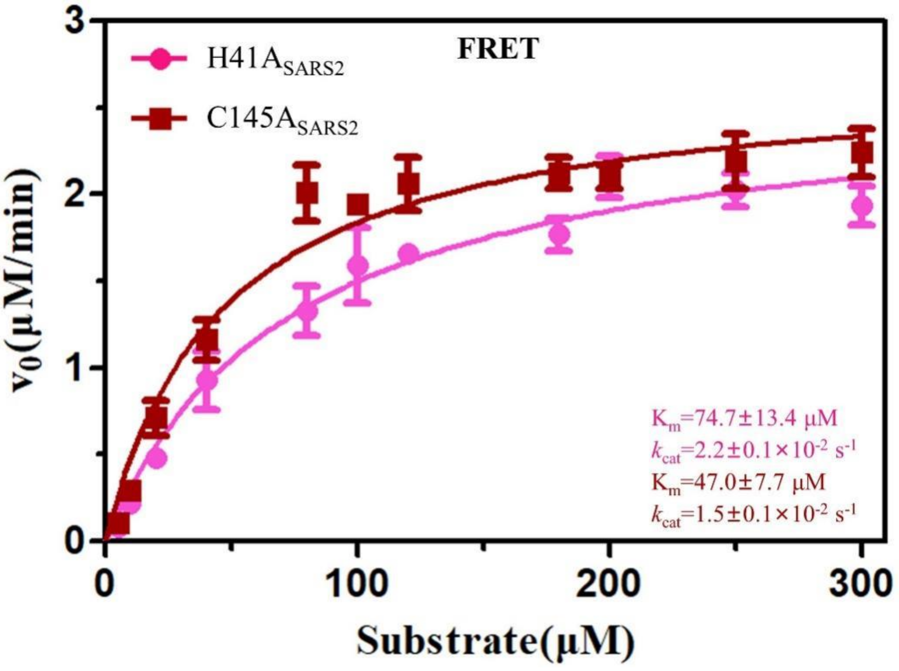
FRET
H41A$_{SARS2}$
C145A$_{SARS2}$
K_m=74.7±13.4 μM
k_{cat}=2.2±0.1×10^{-2} s^{-1}
K_m=47.0±7.7 μM
k_{cat}=1.5±0.1×10^{-2} s^{-1}
v_0(μM/min)
Substrate(μM)
0
1
2
3
0
100
200
300

(b)

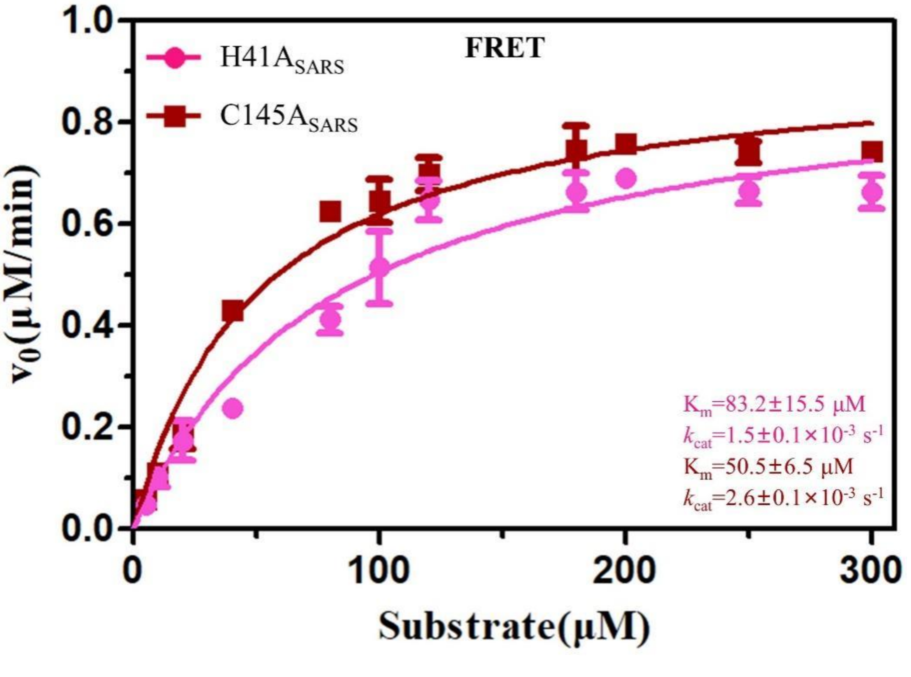
FRET
H41A$_{SARS}$
C145A$_{SARS}$
K_m=83.2±15.5 μM
k_{cat}=1.5±0.1×10^{-3} s^{-1}
K_m=50.5±6.5 μM
k_{cat}=2.6±0.1×10^{-3} s^{-1}
v_0(μM/min)
Substrate(μM)
0.0
0.2
0.4
0.6
0.8
1.0
0
100
200
300

(c)

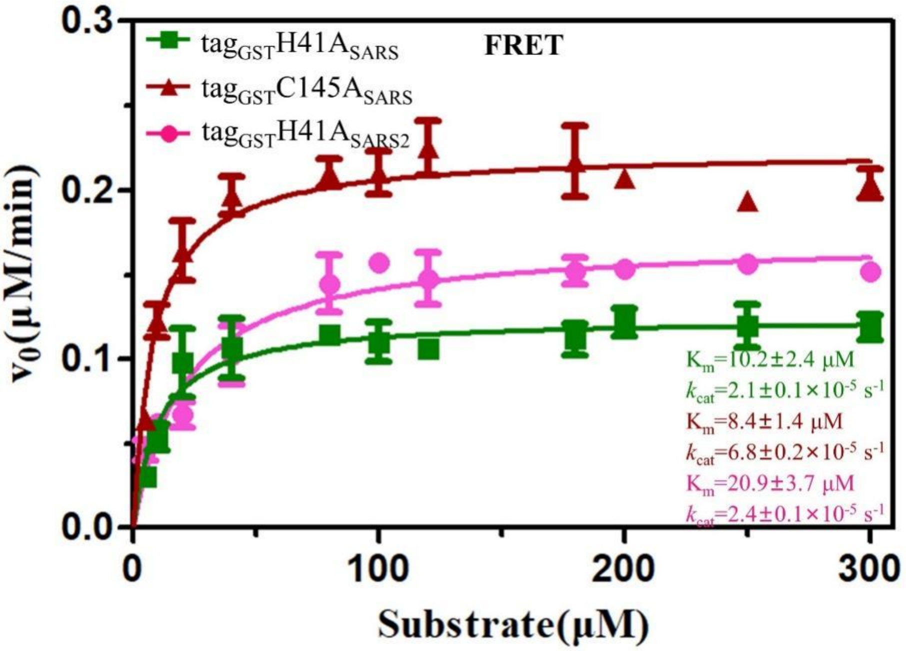

(d)

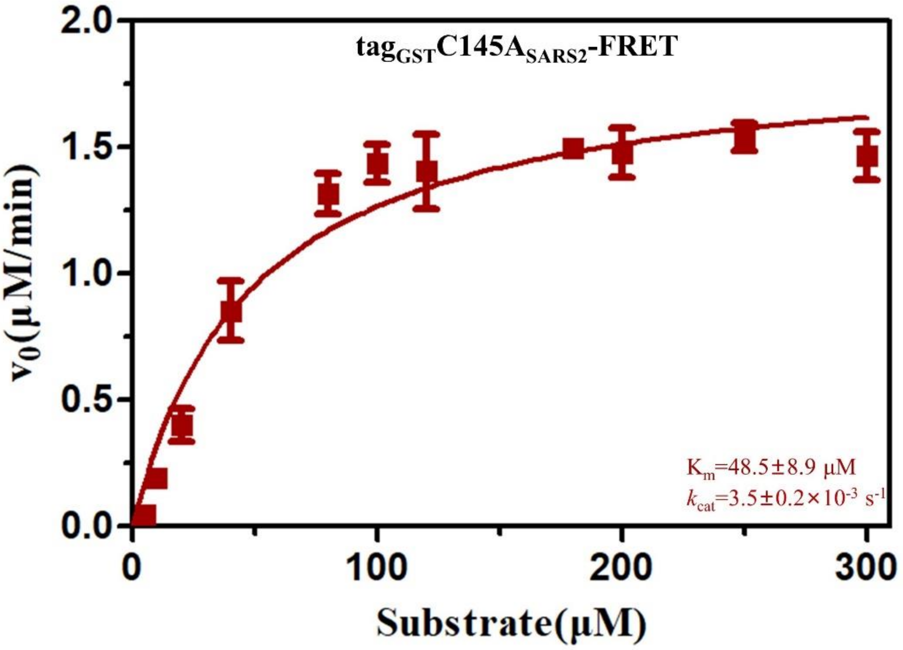

Figure S2. The Michaelis-Menten plot of M^{pro} from mutants. (a) H41A$_{SARS2}$ and C145A$_{SARS2}$ (b) H41A$_{SARS}$ and C145A$_{SARS}$ (c-d) tag$_{GST}$H41A$_{SARS2}$, tag$_{GST}$H41A$_{SARS}$, tag$_{GST}$C145A$_{SARS}$ and tag$_{GST}$C145A$_{SARS2}$. The kinetic parameters were measured using the FRET-based cleavage assay. Values are means ±SEM of at least three independent

experiments performed in triplicates. Curves fit with the Michaelis–Menten equation in GraphPad Prism.

(a)

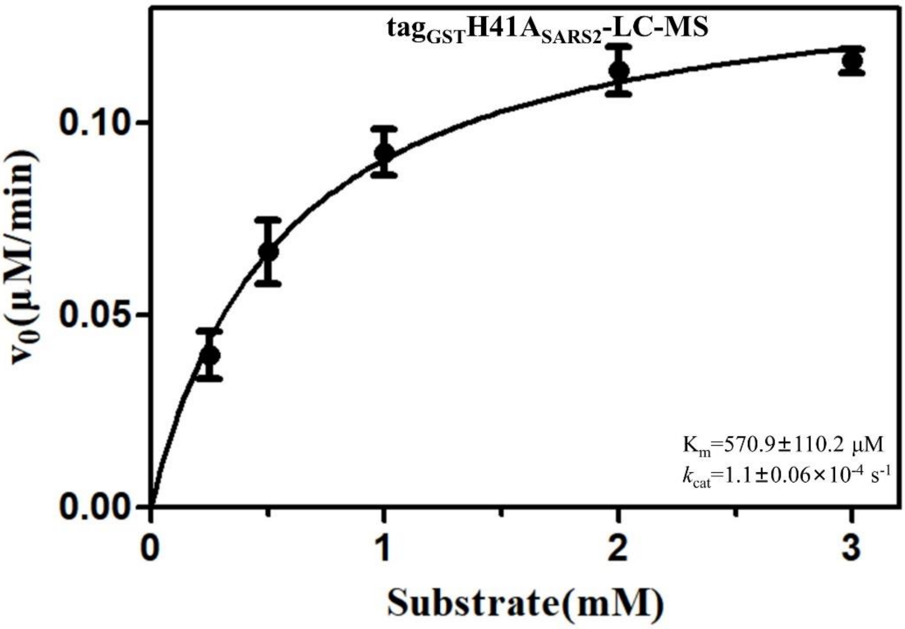

tagGSTH41ASARS2-LC-MS
v0(μM/min)
0.00
0.05
0.10
0
1
2
3
Substrate(mM)
Km=570.9±110.2 μM
kcat=1.1±0.06×10-4 s-1

(b)

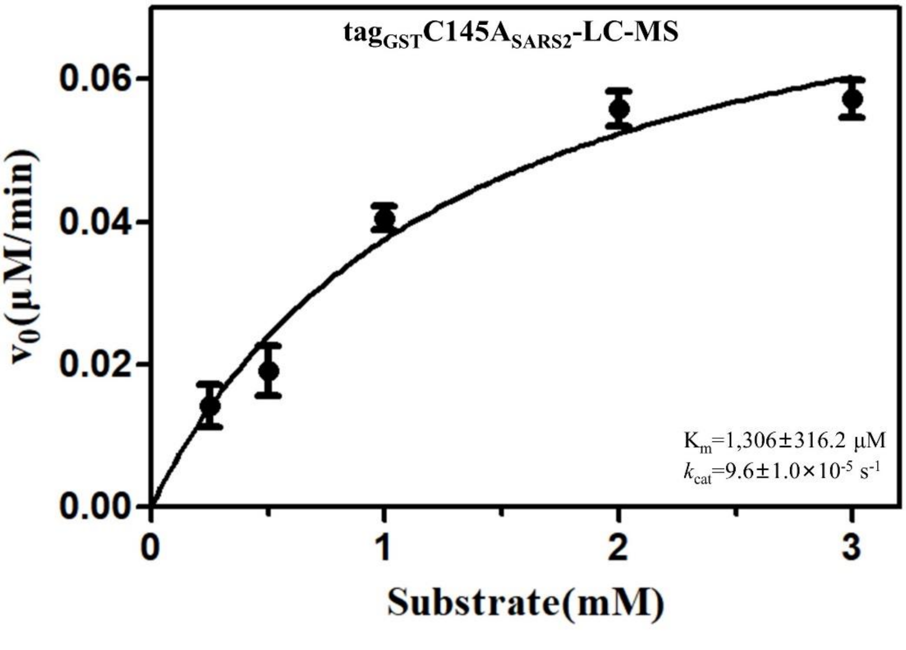

tagGSTC145ASARS2-LC-MS
v0(μM/min)
0.00
0.02
0.04
0.06
0
1
2
3
Substrate(mM)
Km=1,306±316.2 μM
kcat=9.6±1.0×10-5 s-1

(c)

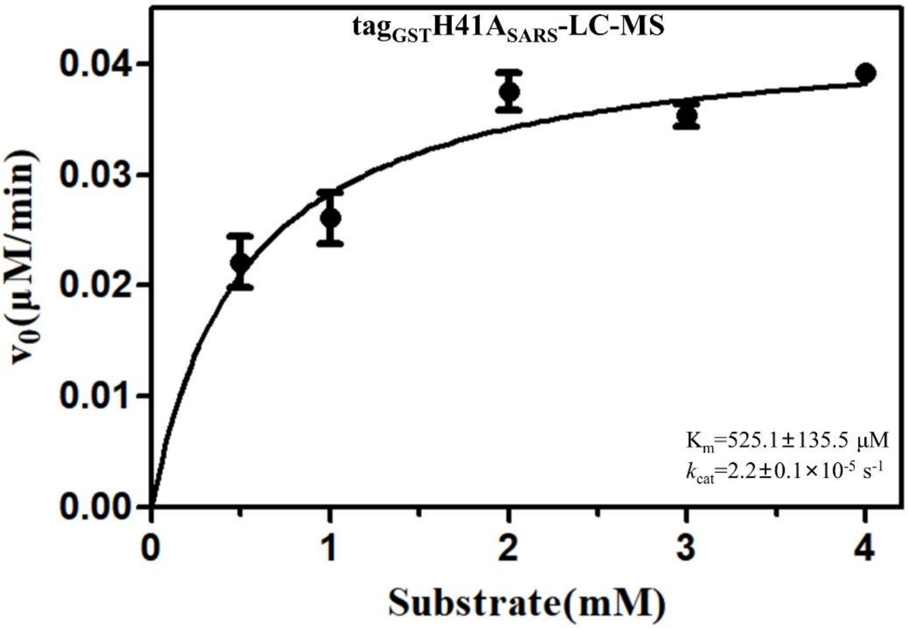

(d)

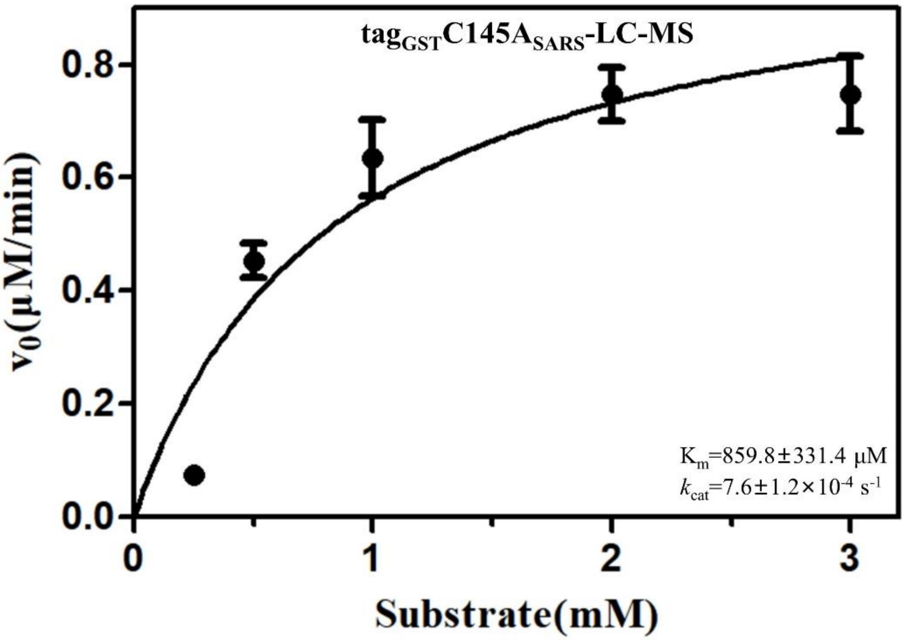

Figure S3. The Michaelis-Menten plot of M^{pro}. (a) $tag_{GST}H41A_{SARS2}$ (b) $tag_{GST}C145A_{SARS2}$ (c)$tag_{GST}H41A_{SARS}$ (d) $tag_{GST}C145A_{SARS}$. The kinetic parameters were measured using LC-MS method. Values are means±SEM of at least three independent experiments performed in triplicates. Curves fit with the Michaelis–Menten equation in GraphPad Prism.

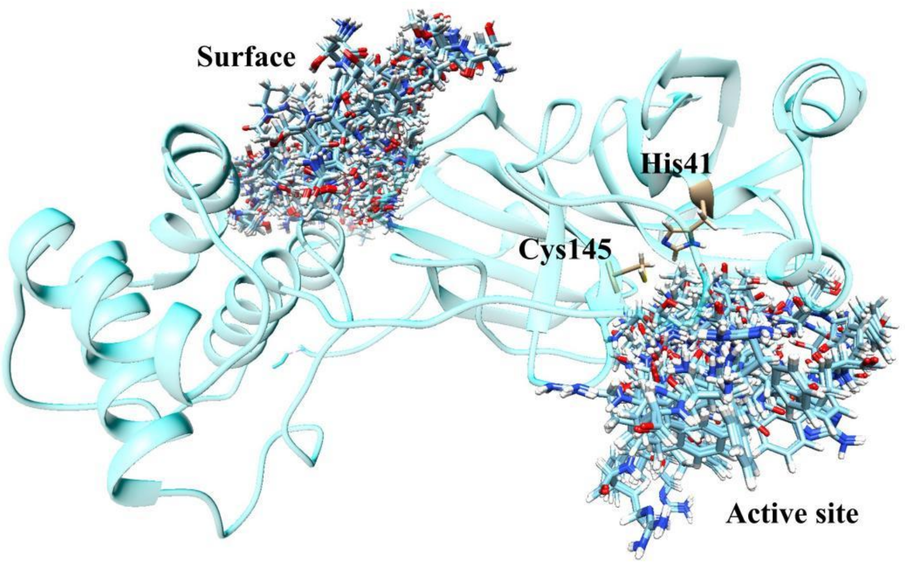

Figure S4. The substrate distribution of binding sites in SARS-CoV-2 was predicted by molecular docking.

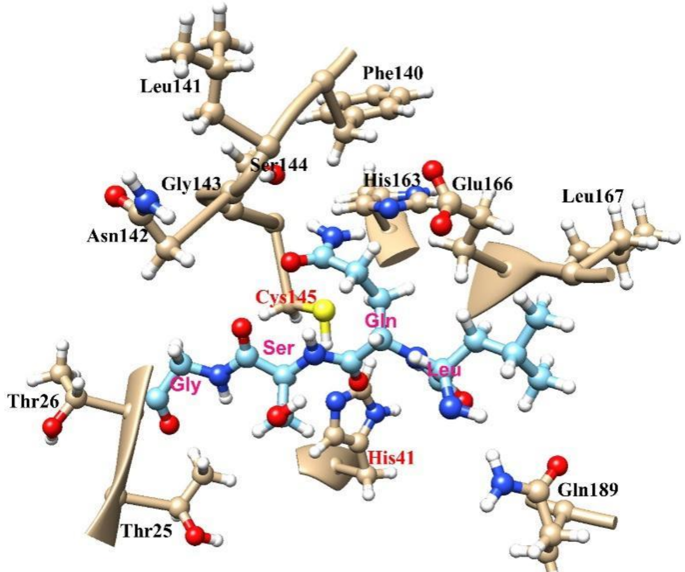

Figure S5. The interactions between M^{pro}_{SARS} and key amino acids (LQSG) in the substrate are predicted by molecular docking.

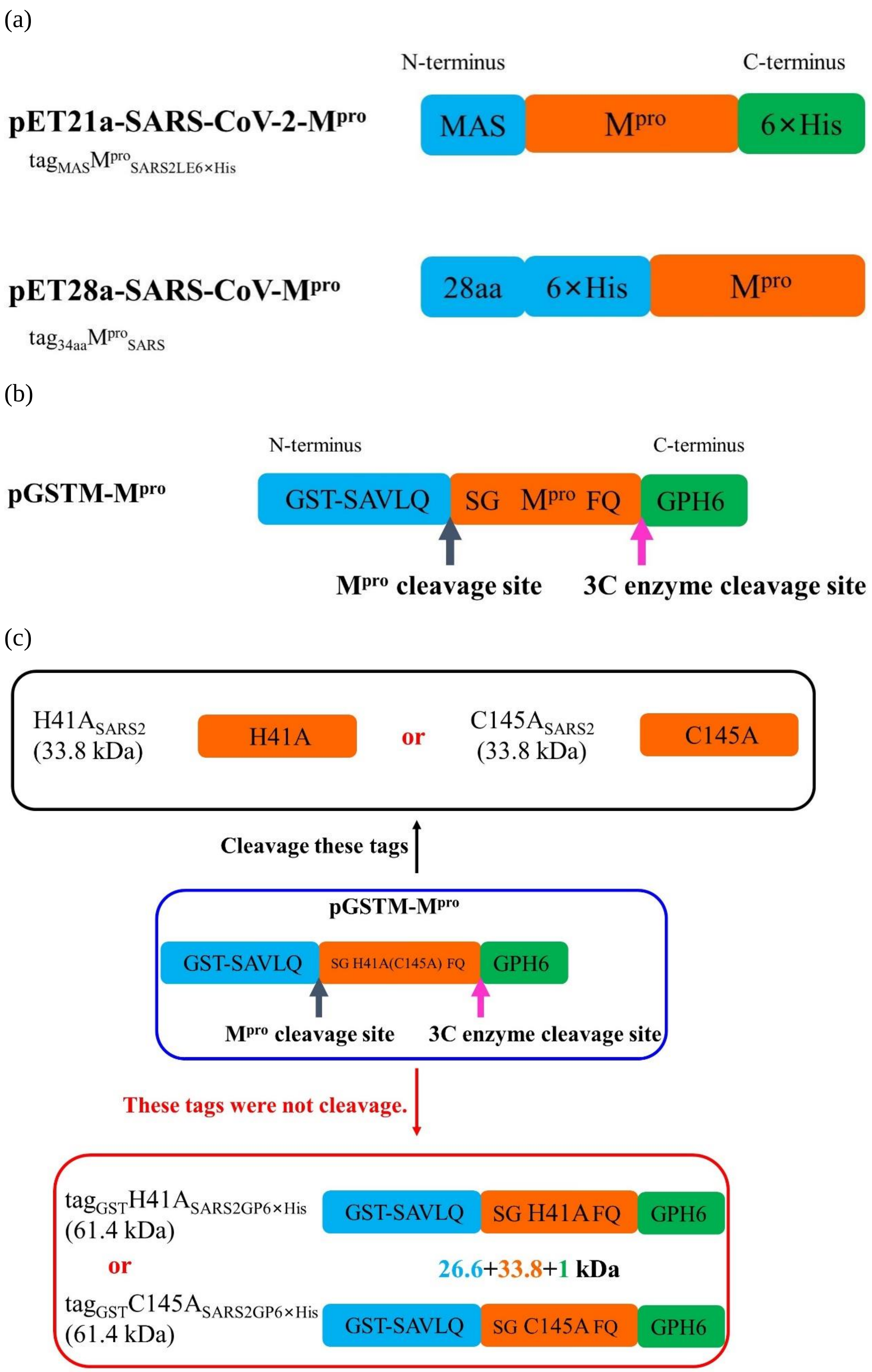

Figure S6. The map of vectors used in the study (a) pET21a-SARS-CoV-2-M^{pro}, and pET28a-SARS-CoV-M^{pro} to obtain M^{pro} with tag. $tag_{MAS}M^{pro}{}_{6\times His}$ = N terminal with

Met-Ala-Ser and C terminal with Leu-Glu-6×His; $tag_{34aa}M^{pro}$ =N terminal with 34 extra amino acids. (b) pGSTM-M^{pro} to obtain native M^{pro} without any tags. (c) Mutants were obtained based on pGSTM-M^{pro} expression system. Before these tags were cleaved, there was a GST tag (218 amino acids, around 26.6 kDa) at the N-terminal and GP6×His tag (8 amino acids, around 1 kDa) at the C-terminus, the molecular weight of mutants with tags was 61.4 kDa. Once these tags were cleaved, the molecular weight of the mutants was 33.8 kDa.

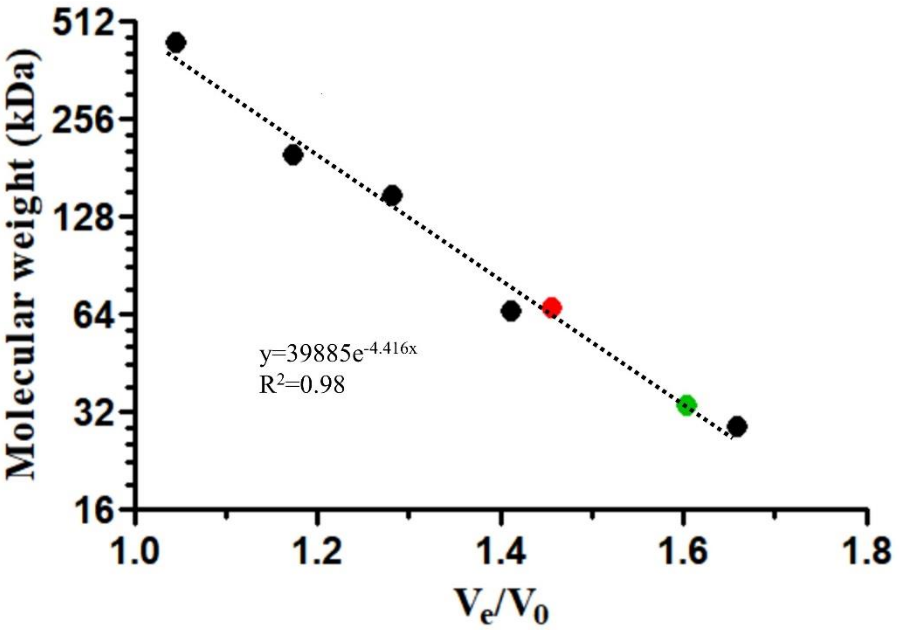

Figure S7. The size exclusion chromatography (SEC) standard curve. The standard proteins were Arbonic Anhydrase, bovine erythrocytes, MW: 29 kDa, Albumin, bovine serum, MW: 66 kDa, Alcohol Dehydrogenase, yeast, MW: 150 kDa, β-Amylase, sweet potato, MW: 200 kDa, Apoferritin, horse spleen, MW: 443 kDa. The red dot and green dot correspond to the dimer (67.6 kDa) and monomer (33.8 kDa) molecular weight of M^{pro} respectively.

(a)

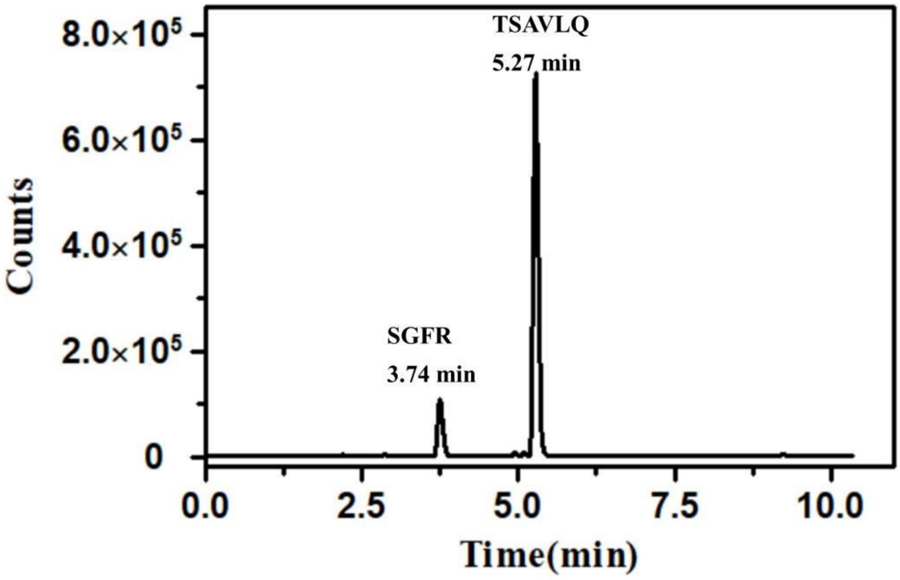

(b)

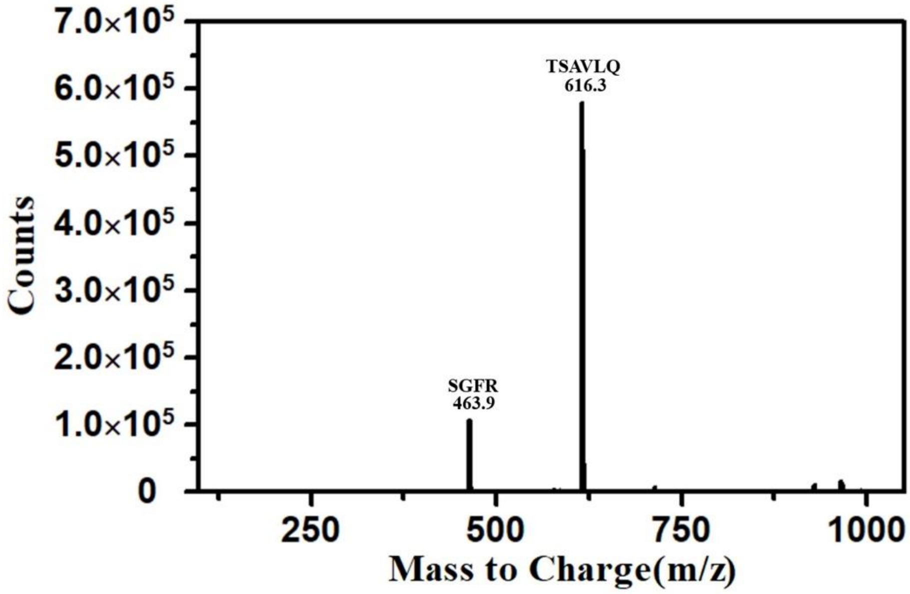

Figure S8. The separation and detection of the product by HPLC/MS. (a) TIC scan of SGFR and TSAVLQ. (b) Product-ion spectra of SGFR and TSAVLQ.

(a)

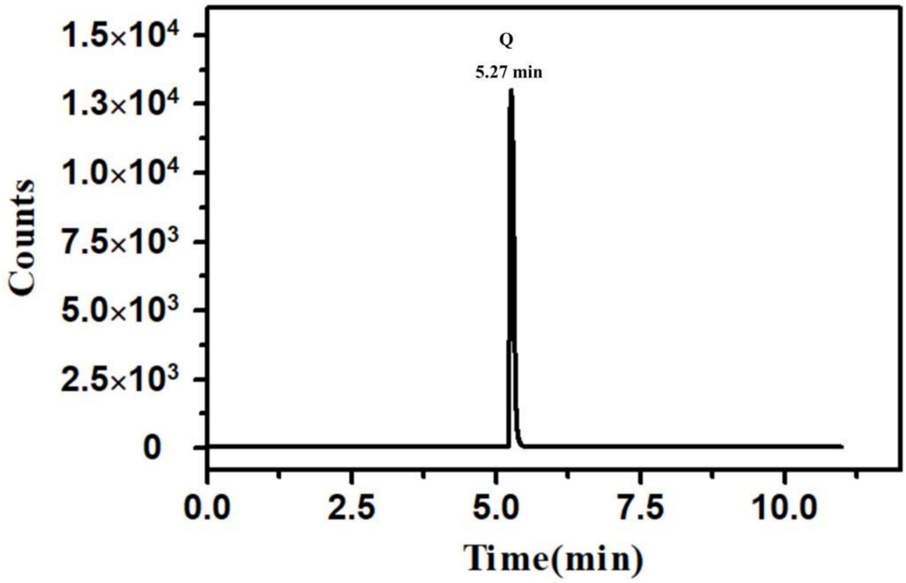

(b)

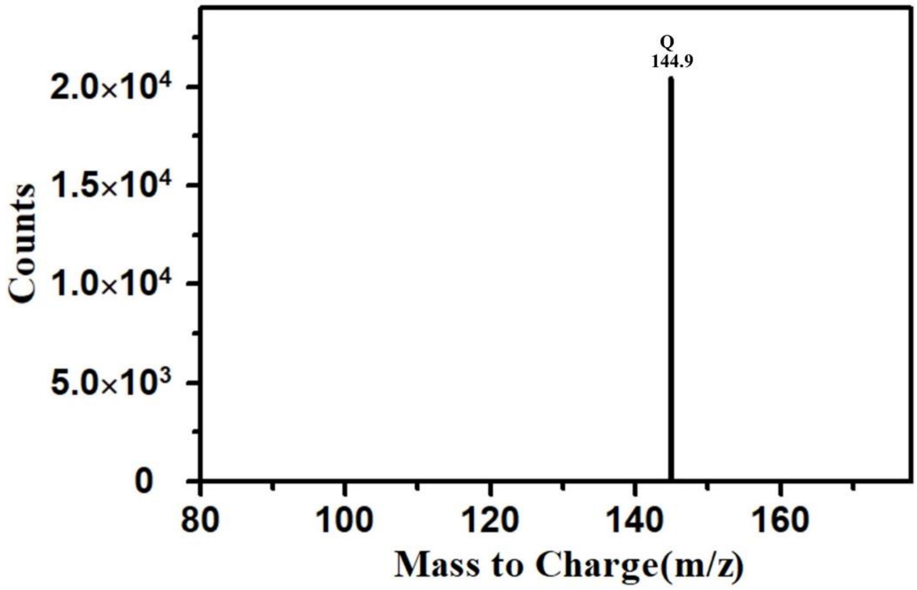

Figure S9. The detection of product TSAVLQ by HPLC/MS. (a) The MRM scan of the product. (b) Precursor→product transition is m/z 616.3→144.9 for TSAVLQ. MRM is a type of tandem mass spectrometry (MS/MS) that involves selecting a precursor ion of interest in the first stage of MS and then fragmenting it to create product ions that are specific to the molecule being analyzed. In the second stage of MS, specific product ions are selected and monitored over time, enabling the detection and quantification of the target molecule.

(a)

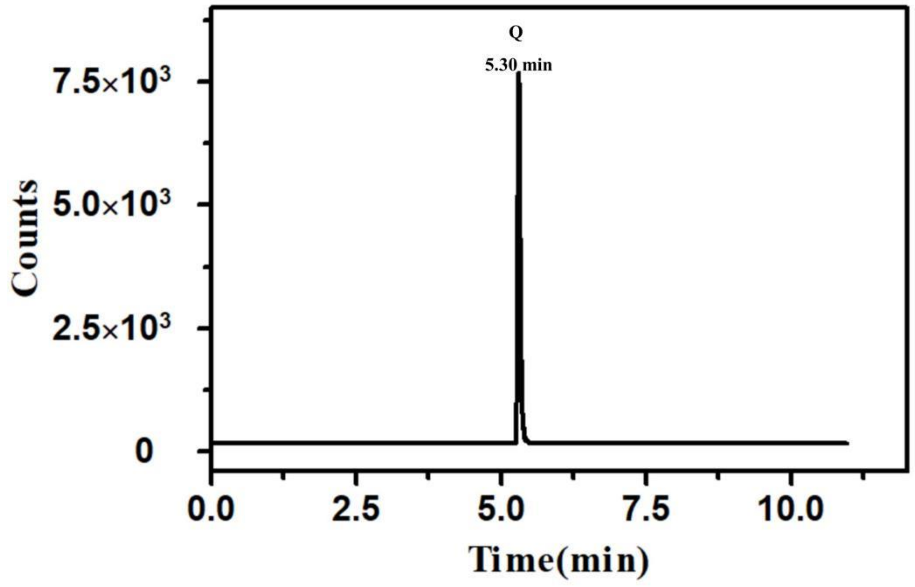

(b)

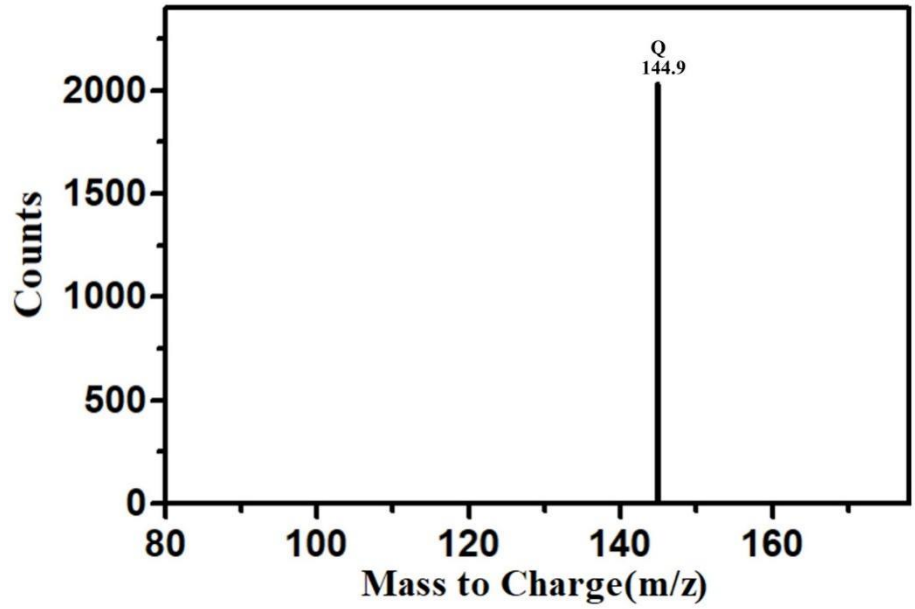

Figure S10. The detection of product TSAVLQ by HPLC/MS. (a) The MRM scan of the product. (b) Precursor→product transition is m/z 616.3→144.9 for TSAVLQ. The reaction was initiated by M^{pro}_{SARS2}.
